# Identification and functional characterization of a *β*-glucosidase from *Bacillus tequelensis* BD69 expressed in bacterial and yeast heterologous systems

**DOI:** 10.7717/peerj.8792

**Published:** 2020-03-30

**Authors:** Ahmad Raza, Ratnasri Pothula, Heba Abdelgaffar, Saira Bashir, Juan Luis Jurat-Fuentes

**Affiliations:** 1 National Institute for Biotechnology and Genetic Engineering (NIBGE), Faisalabad, Pakistan; 2 Pakistan Institute of Engineering & Applied Sciences (PIEAS), Islamabad, Pakistan; 3 Department of Entomology and Plant Pathology, University of Tennessee, Knoxville, TN, United States of America

**Keywords:** β-glucosidase, *Bacillus tequelensis*, Heterologous expression, Glucosidase kinetics, Thermodynamics

## Abstract

**Background:**

The identification and characterization of novel β-glucosidase genes has attracted considerable attention because of their valuable use in a variety of industrial applications, ranging from biofuel production to improved digestibility of animal feed. We previously isolated a fiber-degrading strain of *Bacillus tequelensis* from buffalo dung samples, and the goal of the current work was to identify β-glucosidase genes in this strain. We describe the cloning and expression of a new β-glucosidase gene (*Bteq*βgluc) from *Bacillus tequelensis* strain BD69 in bacterial and yeast hosts. The recombinant Bteqβgluc were used to characterize specificity and activity parameters, and candidate active residues involved in hydrolysis of different substrates were identified through molecular docking.

**Methods:**

The full length *Bteq*βgluc gene was cloned and expressed in *Escherichia coli* and *Pichia pastoris* cultures. Recombinant Bteqβgluc proteins were purified by immobilized metal affinity or anion exchange chromatography and used in β-glucosidase activity assays measuring hydrolysis of *ρ*-nitrophenyl-β-D-glucopyranoside (pNPG). Activity parameters were determined by testing relative β-glucosidase activity after incubation under different temperature and pH conditions. Candidate active residues in Bteqβgluc were identified using molecular operating environment (MOE) software.

**Results:**

The cloned *Bteq*βgluc gene belongs to glycoside hydrolase (GH) family 4 and encoded a 54.35 kDa protein. Specific activity of the recombinant β-glucosidase was higher when expressed in *P. pastoris* (1,462.25 U/mg) than in *E. coli* (1,445.09 U/mg) hosts using same amount of enzyme. Optimum activity was detected at pH 5 and 50 °C. The activation energy (*E*_a_) was 44.18 and 45.29 kJ/mol for Bteqβgluc produced by *P. pastoris* and *E. coli*, respectively. Results from other kinetic parameter determinations, including p*K*_a_ for the ionizable groups in the active site, Gibbs free energy of activation (Δ*G*^‡^), entropy of activation (Δ*S*^‡^), Michaelis constant (*K*_m_) and maximum reaction velocity (*V*_max_) for pNPG hydrolysis support unique kinetics and functional characteristics that may be of interest for industrial applications. Molecular docking analysis identified Glu, Asn, Phe, Tyr, Thr and Gln residues as important in protein-ligand catalytic interactions.

## Introduction

Cellulose in plant biomass is the most abundant carbohydrate on Earth, and there is relevant interest on its potential use in various industries (Godoy et al., 2018; Hassan Hassan Abdellatif et al., 2016). Enzymatic degradation of cellulose into its glucose subunits (saccharification) requires combined activity of a cellulase system comprised of endoglucanases, cleaving internal bonds in the amorphous cellulose, exoglucanases, acting on reducing or non-reducing ends of crystalline cellulose, and β-glucosidases which catalyze the hydrolysis of cellobiose to glucose (Lynd et al., 2002). Efficient β-glucosidase activity is critical to the rate of saccharification, as accumulation of cellobiose activates negative feedback loops affecting cellulase activity (Bhatia, Mishra & Bisaria, 2002). Consequently, β-glucosidases play a pivotal role in various biotechnological processes in the biofuel, food and feed industries (Li et al., 2013; Raza, Bashir & Tabassum, 2019b; Yuksekdag et al., 2017).

Genes encoding β-glucosidases are distributed throughout a wide range of organisms, including microbes, plants, and animals (Chang, Kao & Juang, 2008; Knight & Dick, 2004). Commercially used β-glucosidases are mostly of fungal origin, but production of this enzyme in bacterial systems is of high interest. *Bacillu* s spp. have so far been found as one of the microbial groups with most potential for extracellular production of commercially valuable enzymes (Kim et al., 2009; Saba Khalid, Shakir & Qazi, 2017; Chamoli et al., 2016; Khan & Husaini, 2006; Zahoor et al., 2011). In fact, *Bacillus* isolates from diverse environmental niches have been documented to produce novel β-glucosidase enzymes with unique characteristics amenable to industrial application (Raza, Bashir & Tabassum, 2019c; Raza, Bashir & Tabassum, 2019a; Wu et al., 2018).

During an earlier screening project, we identified *Bacillus tequelensis* strain BD69 as displaying high cellulase activity (Raza, Bashir & Tabassum, 2019a). In present work, we report the cloning and expression of a β-glucosidase gene (*Bteq* β*gluc*) from this strain in bacterial (*Escherichia coli*) and yeast (*Pichia pastoris*) heterologous systems. We used the recombinant enzymes for kinetic and functional characterization of enzyme activities, with optimal activity observed at pH 5 and 50 °C. We also performed molecular docking of Bteqβgluc with *ρ*-nitrophenyl-linked substrates to determine candidate substrate catalytic residues. The unique functional characteristics of the recombinant Bteqβgluc suggests its potential for industrial applications.

**Table 1:** Bacterial strains and vectors used in this study.

**Bacterial strains and vectors**	**Description**	**Source**
**Strains**		
*B. tequilensis* BD69 (Nucleotide accession # MF767893)	Wild type, able to degrade cellulose	Isolated from indigenous buffalo dung samples
DH10β (High efficiency)	Competent cells	New England Biolabs (USA)
*E. coli* BL21 (DE3) pLysS	Expression host	Promega (USA)
*Pichia pastoris* GS115	Expression host	Thermo Fisher Scientific (USA)
**Vectors**		
pET-30a(+)	Expression vector	Novagen (Germany)
pPIC9K	Expression vector	Thermo Fisher Scientific (USA)
pET-Bteqβgluc	Recombinant expression vector, *Bteq*β*gluc* gene cloned into the pET-30a(+) vector	Developed in this study
pPIC-Bteqβgluc	Recombinant expression vector, *Bteq*β*gluc* gene cloned into the pPIC9K vector	Developed in this study

## Materials & Methods

### Strains, vectors, media and culture conditions

The strains and vectors used in this study are listed in Table 1. Briefly, *B. tequilensis* BD69 (GenBank nucleotide accession number: MF767893) is a fiber-degrading strain previously isolated from buffalo dung samples in the Fermentation Technology Group at the National Institute for Biotechnology and Genetic Engineering (NIBGE), Pakistan. Competent *E. coli* DH10 β (high efficiency) cells were used for construction and sequencing of the recombinant expression vectors. For expression of the *β*-glucosidase gene in pET-30a(+) (Novagen, Germany) and pPIC9K (Invitrogen, USA), we used *E. coli* BL21 (DE3) pLysS and *P. pastoris* GS115 (his4, Mut+) cells, respectively.

Cultures of *E. coli* BL21 (DE3) pLysS and DH10 β were grown in Luria-Bertani (LB, 0.5% yeast extract, 1% peptone and 1% NaCl) medium (Sambrook, Russell & Russell, 2001), to which ampicillin (100 µg/mL) or kanamycin (50 µg/mL) were added as required. Isopropyl- β-D-thiogalactoside (IPTG) was used to induce expression in BL21 cells, while *Pichia* transformants were selected for their ability to grow on minimal dextrose (MD) plates (1.34% yeast nitrogen base, 4 × 10 −5% biotin, 2% dextrose, lacking histidine) at 30 °C. Yeast peptone dextrose (YPD; 1% yeast extract, 2% peptone, 2% glucose) agar plates containing geneticin G418 (0.5–2.0 mg/mL) were used for screening of multi-copy *Pichia* colonies. Buffered glycerol-complex medium (BMGY; 1% yeast extract, 2% peptone, 4 × 10 −5% biotin, 1.34% yeast nitrogen base, 1% glycerol, 100 mmol/L potassium phosphate, pH 6.0), and buffered methanol-complex medium (BMMY; 1% yeast extract, 2% peptone, 4 × 10 −5% biotin, 1.34% yeast nitrogen base, 1% methanol, 100 mmol/L potassium phosphate, pH 6.0) were used for expression in *P. pastoris*. Restriction enzymes (fast digest EcoRI and NotI), T4 DNA ligase, DreamTaq Green PCR master mix (2X) and PureLink™ Quick Gel Extraction and PCR Purification Combo Kit were purchased from Thermo Fisher Scientific (USA), whereas the QIAprep spin miniprep kit was procured from Qiagen (USA). All other chemicals used were of analytical grade.

### DNA extraction, primer design and gene amplification

A single colony of *B. tequilensis* BD69 was inoculated into sterile LB broth and after overnight incubation at 37 °C, the DNA was extracted from bacterial cells with a conventional phenol-chloroform-isoamyl alcohol method (Sambrook, Russell & Russell, 2001). The presence of DNA was confirmed by 1% agarose gel electrophoresis. NanoDrop was used to quantify the purity and concentration of DNA.

Specific primers were designed to amplify the *Bteq* β*gluc* gene based on a multiple sequence alignment to identify conserved sequences among β-glucosidase gene sequences retrieved from the NCBI database (Magrane & UniProt Consortium, 2011). The designed PCR primers were confirmed *in silico* (San Millán et al., 2013). The forward (GAATTCATGACAAAAGGATTGAAGATTGTAAC) and reverse (GCGGCCGCTTACGCTTCAATTTTGTTGAAAAACTGC) primers containing EcoR1 and Not1 restriction sites (underlined), respectively, were used for amplification of *Bteq* β*gluc* gene from *B. tequilensis*. DreamTaq Green PCR master mix (2X) was used as per manufacturer’s instructions with thermocycling conditions as follows: initial denaturation 95 °C for 5 min, followed by 30 cycles of 95 °C for 1 min, 60 °C for 30 s, 72 °C for 1 min, and final elongation at 72 °C for 10 min. The amplified product was checked for size and purity on a 1% (w/v) agarose gel.

### Construction of recombinant plasmids and sequence analysis

The PCR amplicon from the reaction detailed above and expression vectors pET-30a(+) and pPIC9K were restricted by fast digest EcoRI and NotI enzymes, and then ligated by T4 DNA ligase to generate the pET-Bteqβgluc and pPIC-Bteqβgluc expression constructs, respectively. Constructs were chemically transformed into DH10 β *E. coli* cells according to the manufacturer’s instructions. Recombinants were selected on LB plates containing kanamycin (50 µg/mL) and ampicillin (100 µg/mL) for pET-30a(+) and pPIC9K, respectively. Plasmid extraction was done using the QIAprep spin miniprep kit, and positive recombinant clones were identified by double digestion (Fig. S1) and colony PCR followed by sequencing (Genomics Core, University of Tennessee, Knoxville, USA). Sequencing results were analyzed using DNAMAN 9.0 (http://dnaman.software.informer.com/9.0) and searching the NCBI database using BLAST (McGinnis & Madden, 2004). The β-glucosidase open reading frame (ORF) was predicted by ORF Finder (http://www.ncbi.nlm.nih.gov/gorf/gorf.html) and used to confirm proper reading frame in recombinants. A BLASTx search was also performed to retrieve homologous proteins, translated amino acid sequences were obtained using the EXPASY translate tool (Gasteiger, 2003). The signal peptide for the β-glucosidase gene was predicted using SignalP 4.0 (http://www.cbs.dtu.dk/services/SingnalP/). Putative N-glycosylation sites were located using the NetNGlyc program 1.0 (http://www.cbs.dtu.dk/services/NetNGlyc/). Physicochemical properties of the recombinant Bteqβgluc were identified using Protparam (http://au.expasy.org/tools/protparam.html). The conserved domains of the Bteqβgluc were identified by InterProScan (http://www.ebi.ac.uk/Tools/InterProScan/).

### Transformation and expression

Bacterial expression was performed by transforming *E. coli* BL21 (DE3) pLysS (Promega) cells with the pET-Bteqβ gluc vector. One mL of an overnight culture of the recombinant bacteria in LB broth containing 50 µg/mL kanamycin was transferred to 50 mL of the same medium and incubated at 37  °C in a shaking incubator until OD600 = 0.4–0.8. The maximum expression conditions of recombinant Bteqβgluc were optimized by performing induction at different temperatures (16−37 °C), IPTG concentrations (0.3–1 mM) and time (2–16 h) of induction. Cells were harvested by centrifugation at 6,000 × *g* for 10 min, and pellets were re-suspended in 50 mM Tris–HCl (pH 7.5). Collected cells were disrupted by sonication (Fisherbrand™ Q500 Sonicator, 5 s on 5 s off, 7 pulses) and the sample cleared from debris by centrifugation at 12,000 × *g* for 30 min. Expression and solubilization of Bteqβgluc were confirmed by SDS-10% PAGE according to Laemmli (1970).

Expression of Bteqβgluc in *P. pastoris* was started by first linearizing pPIC-Bteqβgluc with SalI and then transforming it into *P. pastoris* GS115 (Invitrogen) by electroporation (1,500 V, 25 µF and 200 Ω). Transformed cells were grown on MD plates at 30 °C for 4 days and then screened on YPD plates containing G418 (0.5–2 mg/ml) for multi-copy insertion of vector. Genomic DNA of *P. pastoris* was extracted using the Yeast DNA Extraction Kit (Thermo Fisher Scientific, USA) for confirmation of positive transformed colonies by PCR with AOX1 and gene specific primers.

Ten confirmed transformants were grown in 10 mL of BMGY medium at 30 °C for 24–36 h at 250 rpm in order to identify the transformant with the relative maximal expression levels. The selected transformant (based on β-glucosidase activity assay) was grown in 300 mL of BMGY medium for 24 h at 250 rpm and 30 °C. The cells were harvested at 3,000× g in a swinging bucket rotor at 4 °C for 5 min, and then grown to 500 ml of BMMY at 30 °C and 250 rpm for production of enzyme for enzyme assays.

### Purification of recombinant proteins

The chromatograms for purification of Bteqβgluc are presented in the supplementary files (Fig. S2). Recombinant Bteqβgluc produced in *E. coli* was purified by immobilized metal affinity chromatography (IMAC) using a HisTrap HP 5 ml column connected to an AKTA Pure FPLC system (GE Healthcare, Sweden). The mobile phase was 50 mM Tris–HCl (pH 7.5), 300 mM NaCl and 20 mM imidazole with a flow rate of five mL/min. Elution was performed on a gradient of imidazole (20 to 500 mM over 10 column volumes) at a flow rate of 3 mL/min.

Anion exchange chromatography was used for purification of recombinant Bteqβgluc produced in yeast cultures. Protein samples were loaded on a HiTrap Q-FF column (five mL) pre-equilibrated in 50 mM Tris–HCl (pH 7.5), and elution was performed on a linear gradient of NaCl (0–1 M, 10 column volumes) at a flow rate of 3 mL/min.

Purified Bteqβgluc was concentrated and desalted against Milli Q water using Pierce Concentrators (9K MWCO, Thermo Fisher Scientific, USA) at 4 °C. Samples were then analyzed on 10% SDS-PAGE and by Western blotting as detailed below.

### Western blotting and electrophoretic analysis

Purified recombinant Bteqβgluc (50 µg) was resolved in SDS-10% PAGE and stained with Protoblue stain (National diagnostics, USA) or electrotransferred at 60 V for 1 h in Towbin’s transfer buffer (192 mM glycine, 25 mM Tris, 0.1% SDS, 20% methanol) to nitrocellulose membranes (0.45 µm, Thermo Fisher Scientific, USA). After blotting, the filter was blocked in 1X PBS, 0.1% Tween-20, 3% BSA for 45 min with continuous shaking, and then incubated for 1 h at room temperature in washing buffer (1X PBS, 0.1% Tween-20, 0.1% BSA) containing anti-6X-His tag monoclonal antibody (1:2,000 dilution). After washing once for 15 min and 5 times for 10 min each with washing buffer, the filter was developed using enhanced chemoluminescence (SuperSignal™ West Pico Chemiluminescent Substrate, Thermo Scientific, USA) and imaged in an Imager 6000 (GE Healthcare).

### *β*-glucosidase assay

Concentration of purified Bteqβgluc was determined using the Qubit Protein Assay Kit (Invitrogen, USA). Activity of β-glucosidase in purified samples was determined by measuring hydrolysis of *ρ*-nitrophenyl- *β*-D-glucopyranoside (pNPG) as previously described (Cai, Buswell & Chang, 1998; Shi et al., 2017), with minor modifications. Briefly, reaction mixtures (150 µL) containing 10 mM pNPG in 50 mM acetate buffer (pH 5.0) were mixed with an appropriately diluted enzyme solution (150 µg total protein) and incubated at 50 °C for 30 min as previously optimized. The reactions were stopped by adding 150 µL of 1 M Na_2_CO_3_, and *ρ*-nitrophenol produced was detected at 400 nm in a spectrophotometer. One unit of β-glucosidase was defined as the amount of enzyme liberating 1 µmol of *ρ*-nitrophenol per minute under the assay conditions. Activity of β-glucosidase (amount of substrate converted by the enzyme per unit time) is expressed as units per min (U/min), while specific activity (defined as Bteqβgluc activity per milligram of total enzyme) values are presented as units per milligram (U/mg).

### Functional characterization of Bteqβgluc

Stability of purified recombinant Bteqβgluc was tested by incubating the enzyme under different temperature and pH conditions and then assaying the relative activity. The optimal temperature for enzyme activity was determined by testing a temperature range from 30 °C to 80 °C. Whereas, thermal-stability of pET-Bteqβgluc and pPIC-Bteqβgluc was determined by pre-incubating enzyme at different temperature ranging from 50−80 °C. The effect of temperature on the rate of reaction was expressed in terms of temperature quotient (*Q*_10_), which is the factor by which rate increases due to rise in temperature by 10 °C (Reyes, Pendergast & Yamazaki, 2008). The optimum pH for the recombinant enzyme was determined by testing *β*-glucosidase activity over a pH range from 2–12 in increments of 2 pH units at 50 °C. While, pH stability was determined by pre-incubating Bteqβgluc at different pH values (2–12) at 50 °C for 1 h.

The effect of incubation time on enzyme activity was tested by incubating the enzyme with the substrate (pNPG) for different time intervals (10–100 min) and then determining the amount of product (*ρ*-nitrophenol) released. Kinetic parameters, including the Michaelis constant (*K*_m_), maximum reaction velocity (*V*_max_), turn over number (*k*_cat_), and catalytic efficiency (*k*_cat_/*K*_m_) were estimated by determining the β-glucosidase activity at 50 °C against different pNPG concentrations ranging from 2–20 mM with a constant enzyme concentration. Both *K*_m_ and *V*_max_ were calculated from Michaelis–Menten equation using non-linear adjustment and Lineweaver-Burk double reciprocal plots (Lineweaver & Burk, 1934) using the following equation:

\begin{eqnarray*}1/\mathrm{\upsilon }=(1/{V}_{\mathrm{max}})+({K}_{\mathrm{m}}/{V}_{\mathrm{max}})1/[S] \end{eqnarray*}

Where υ is reaction velocity (reaction rate), *V*_max_ is the maximum reaction velocity, *K*_m_ isthe Michaelis–Menten constant, and [S] is the substrate concentration.

Thermodynamic parameters for processing of the pNPG substrate were calculated by rearranging Eyring’s absolute rate equation derived from transition state theory (Eyring & Stearn, 1939) and Arrhenius plot using equations as described by Siddiqui et al. (1997a):

\begin{eqnarray*}{k}_{\mathrm{cat}}=(({k}_{\mathrm{B}}\times T)/h)e\wedge (((-\Delta H\ddagger )/RT))e\wedge ((\Delta S\ddagger /R)) \end{eqnarray*}

Where, *k*_B_ is Boltzmann’s constant = 1. 38 × 10^−23^ J/K, *T* is absolute temperature (K), *h* is Plank’s constant = 6.626 ×10^−34^ J s, *R* is gas constant = 8.314 J/K/mol, Δ*H*‡ is enthalpy of activation, and Δ*S*‡ is entropy of activation. The p*K*_a_1 and p*K*_a_2 values of ionizable groups of active site residues were calculated using Dixon plots (Dixon & Webb, 1979). The statistical significance between kinetic parameters and Bteqβgluc expressed in *E. coli* and *P. pastoris* was determined by using two-way analysis of variance (ANOVA) using statistical software (IBM SPSS Statistics 25).

### Molecular docking

We used homology modeling in the SWISS-MODEL workspace (Arnold et al., 2005) to construct the predicted 3D structure for the recombinant Bteq βgluc. The quality of models was evaluated using Ramachandran’s plot on PDBSum as well as QMEAN scoring function and SAVES Package (PROCHECK, and VERIFY3D). The best model was chosen for docking. The 3D protonation of the selected model was carried out using the Molecular Operating Environment (MOE) software. The protein structure was minimized using MMFF94X ForceField. The 3D structures of *ρ*-nitrophenyl-linked substrates, *ρ*-nitrophenyl- β-D-1,4-glucopyranoside (pNPG), 4-methylumbelliferyl- β-D-glucopyranoside (MUG), and saligenin β-D-glucopyranoside (salicin) were downloaded from the PubChem database (https://pubchem.ncbi.nlm.nih.gov/) and minimized using MOE MMFF94S ForceField. MOE was used for enzyme-substrate docking and subjected to hydrogen bonding analysis. The LigX feature of MOE was used to find interactions among Bteqβ gluc and ligands. Protein ligand interaction fingerprints (PLIF) was used to determine common active residues involved in catalysis.

## Results

### Cloning and bioinformatics analysis of the Bteqβ gluc gene

**Figure 1:**
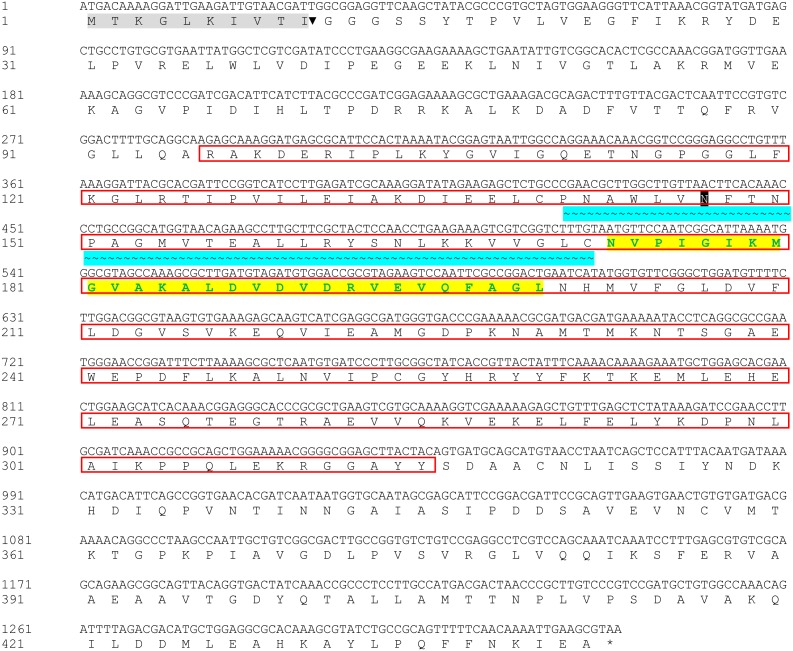
Nucleotide and deduced amino acid sequences of Bteq*β*gluc. Putative signal peptide (10 amino acids) are shown with gray shading followed by cleavage site (▾); predicted glycosylated amino acids (N) shown in black boxes with white text; sugar binding site is red boxed; with cyan background presenting glycoside hydrolase family 4 (GHF4) signature; amino acids with bold text and yellow background present divalent metal binding site; asterisk (*) indicates the transcriptional stop codon.

The Bteqβgluc gene (1,329 bp) was amplified from the genome of *B. tequelensis* BD69 by PCR using sequence specific primers and submitted to GenBank with accession number MK774666. Sequence analysis revealed that this β-glucosidase gene encodes a protein of 443 amino acids including a putative signal peptide of 10 amino acids at the N-terminus. The protein, which we named Bteqβgluc, contains a typical glycoside hydrolase (GH) family 4 signature between residues 141 and 172 and was predicted to contain a single potential N-glycosylation site at position Asn171. The protein also includes a sugar binding site (amino acids 96 to 315) and a divalent metal binding site (amino acids 172 to 202), as shown in Fig. 1.

**Figure 2:**
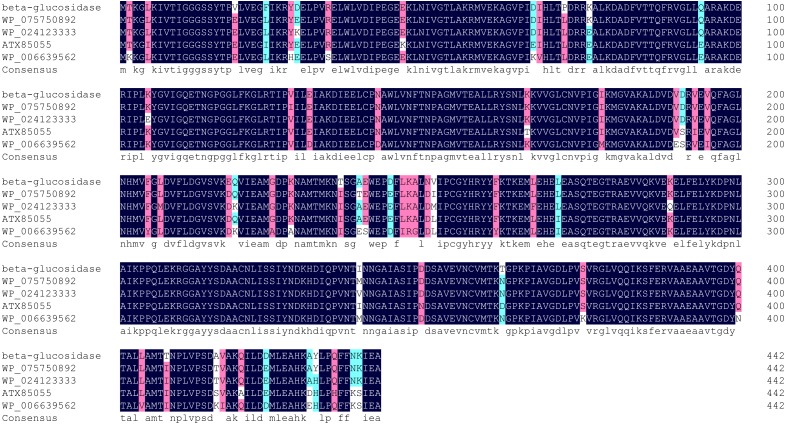
Multiple sequence alignment of Bteqβgluc with four bacterialβ-glucosidases from GH family 4. Alignment is based on the primary structural features determined with BLAST in GenBank. Abbreviations: WP_046160997 (*Bacillus sp.* CMAA 1185); WP_075750892 (*Bacillus licheniformis*); ATX85055 (*Bacillus velezensis*) and WP_006639562 (*Bacillus sonorensis*). Black color indicates fully conserved residues, pink color indicates high scoring groups fully conserved, cyan color indicates moderate scoring groups fully conserved, while white color indicates weaker scoring group is fully conserved.

Multiple sequence alignment showed highest homology (98%) between Bteqβgluc and β-glucosidases from *Bacillus sp.* CMAA 1185 (Fig. 2). The predicted 3D structure for Bteqβgluc showed structural homology with β-glucosidases from *B. stearothermophilus* (79.91%) and *Geobacillus stearothermophilus* (79.50%).

**Figure 3:**
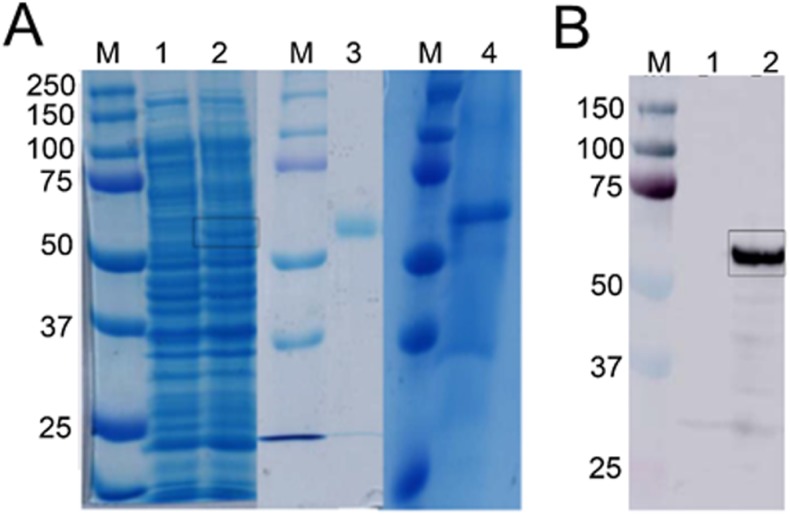
Detection of recombinant Bteqβgluc produced in bacterial and yeast expression cultures. (A) SDS-PAGE gel of recombinant Bteqβgluc produced in *E.coli* (lanes 1–3) or *P. pastoris* (lane 4). M: molecular weight markers, lane 1: non-induced pET-Bteqβgluc sample; lane 2: IPTG induced pET-Bteqβgluc sample, lane 3: purified recombinant Bteqβgluc from *E. coli* cultures; lane 4: purified recombinant Bteqβgluc from *P. pastoris* cultures. (B) Western blot detection of purified His-tagged Bteqβgluc from *E. coli* cultures. M: molecular weight markers; Lane 1: non-induced pET-Bteqβgluc samples; Lane 2: IPTG induced pET-Bteqβgluc sample.

### Expression and purification of Bteq*β* gluc

Expression of Bteqβgluc in *E. coli* and *P. pastoris* resulted in recombinant proteins of the predicted molecular weight (54.34 kDa) in SDS-PAGE (Fig. 3A) and Western blotting (Fig. 3B). In the bacterial expression system, Bteqβgluc was produced as inclusion bodies even under different tested culture conditions (temperature 16−37 °C, pH 5-11). Solubilization of inclusion bodies in 8 M urea followed by refolding resulted in comparatively much lower levels of purified soluble Bteqβgluc protein isolated in *E. coli* compared to *P. pastoris*. Relative Bteqβgluc yields were 1.75 mg/L of culture for *P. pastoris* and 0.68 mg/L of culture for *E. coli*. In addition, the maximum activity (*V*_max_) using same amount (150 µg) of recombinant Bteqβgluc was also higher when the enzyme was purified from *P. pastoris* (1,462.25 U/mg) compared to *E. coli* (1,445.09 U/mg).

**Figure 4:**
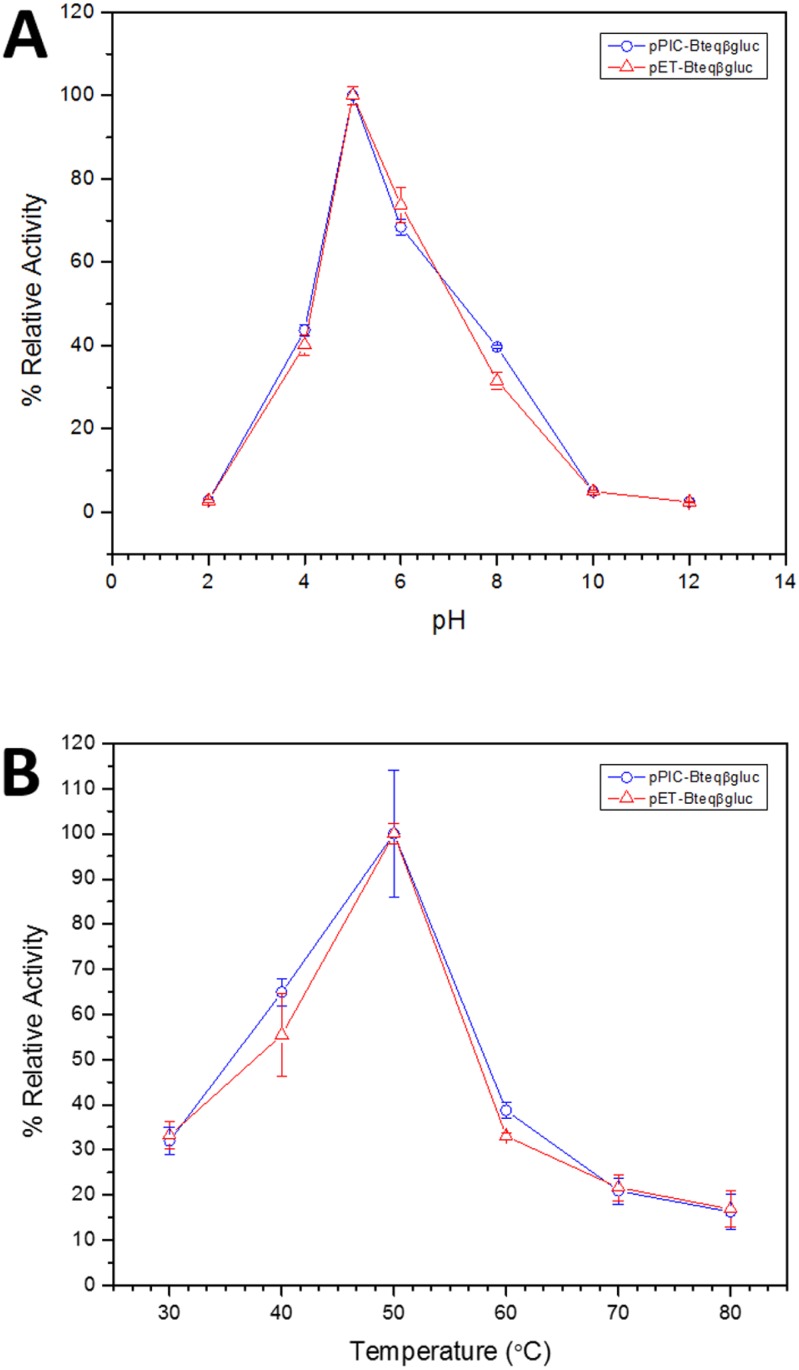
Effect of pH (A) and temperature (B) on the relative activity of Bteqβgluc against pNPG as substrate, when the enzyme is produced in cultures of *E. coli* (pET-Bteqβgluc) or *P. pastoris* (pPIC-Bteqβgluc).

### Kinetics and Functional characterization of recombinant Bteq*β*gluc

**Figure 5:**
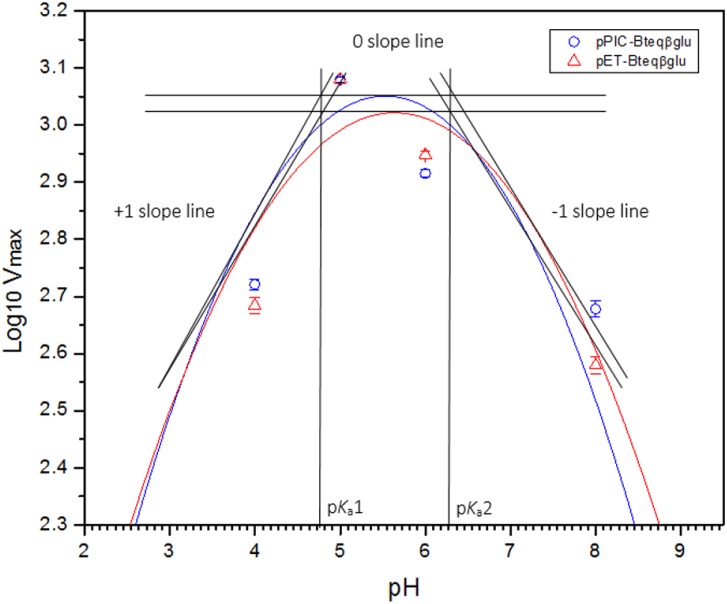
Dixon plot of the activity of Bteqβgluc against pNPG as substrate. Dixon plot of the activity of Bteqβgluc was purified from *E. coli* (pET-Bteqβgluc) or *P. pastoris* (pPIC-Bteqβgluc) cultures. The plot was obtained by determining the *V*_max_ for reactions at different pH and 50 °C for the determination of p*K*a values.

**Figure 6:**
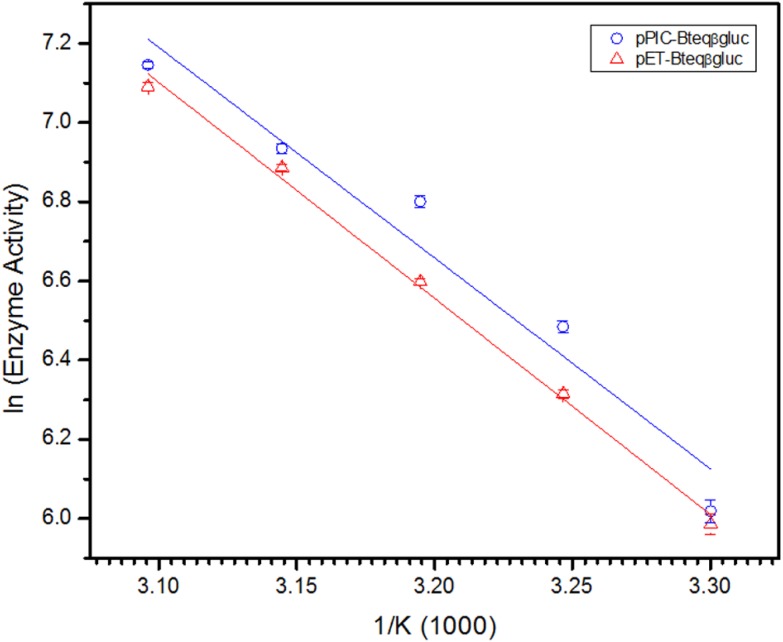
Arrhenius plot for for Bteqβgluc for the determination of activation energy (*E*_*a*_) and enthalpy of activation (Δ*H*‡) against pNPG as substrate. Bteqβgluc was produced in cultures of *E. coli* (pET-Bteqβgluc) or *P. pastoris* (pPIC-Bteqβgluc).

**Figure 7:**
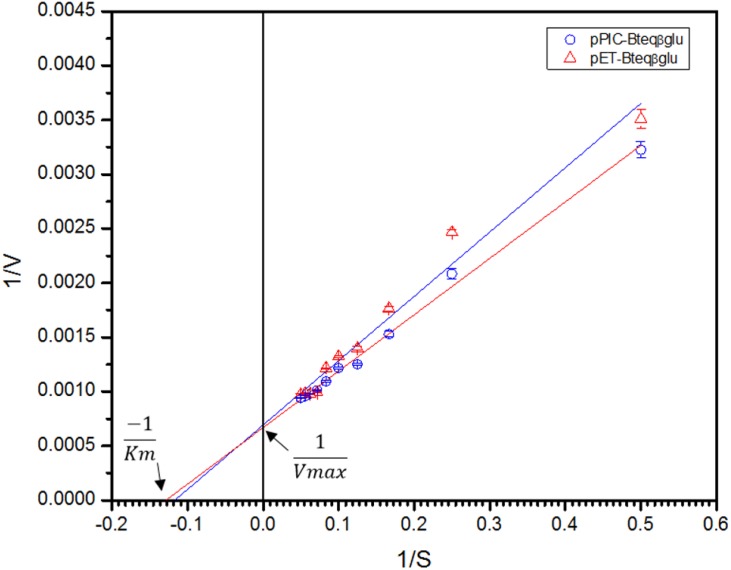
Lineweaver-Burk double reciprocal plot for Bteqβgluc using pNPG as substrate. Bteqβgluc was produced in cultures of *E. coli* (pET-Bteqβgluc) or *P. pastoris* (pPIC-Bteqβgluc).

Purified Bteqβgluc proteins from bacterial and yeast cultures showed statistically significant (*p* < 0.05) difference in specific activities (Table S1). The activity of Bteqβgluc was examined over a range of pH from 2-12 at 50 °C, with optimum activity observed at pH 5 (Fig. 4A). The enzyme was stable at a range of pH from 4–8 for 1 h (Fig. S5A). Enzyme activity increased with increasing temperature from 30 °C and reached a maximum at 50 °C, after which higher temperatures drastically reduced activity suggesting denaturation (Fig. 4B). The enzyme was stable at 50 °C for 180 min and showed degradation above when incubated above 50 °C (Figs. S5B and S5C). Dixon analysis was carried out to evaluate the p*K*_a_ of ionizable groups in active amino-acid residues for pNPG hydrolysis at 50 °C. The 0 slope (on the top of bell shaped curve), +1 and −1 slope lines were drawn. The intersection points of +1 and −1 slope lines on the 0 slope line represented the p*K*_a_1 and p *K*_a_2 as 4.8 and 6.4, respectively (Fig. 5). The activation energy (*E*_a_) was observed to be 44.18 and 45.29 kJ/mol for pPIC-Bteqβgluc and pET-Bteqβgluc, respectively (Fig. 6), while temperature quotient for Bteqβgluc was 1.66. Variables of Δ*H*‡, Δ*G*‡ and Δ*S*‡ were calculated as 41.69 kJ/mol, 57.88 kJ/mol and −54.29 kJ/mol/K, respectively for pPIC-Bteqβgluc. While Δ*H*‡, Δ*G*‡ and Δ*S*‡ for pET-Bteqβgluc were calculated as 42.82 kJ/mol, 57.85 kJ.mol and −50.46 kJ/mol/K, respectively. Free energy of transition state binding (Δ*G*‡_*E*−*T*_) and free energy of substrate binding (Δ*G*‡ _*E*__-__*S*_) were −10.13 kJ/mol and 4.97 kJ/mol, respectively. The *K*_m_ and *V*_max_ values for pPIC-Bteqβgluc and pET-Bteqβgluc were determined through Lineweaver-Burk and Michaelis–Menten plots for the hydrolysis of pNPG at 50 °C. Results obtained for pPIC-Bteqβgluc were similar for both methods, with *K*_m_ values between 7.43 and 7.81 mM and *V*_max_ values between mM and 1,462.25 and 1,505.00 U/mg, from Linewaver-Burk and Michealis-Menten plots, respectively (Fig. 7 and Fig. S6). Similarly, *K*_m_ values for pET-Bteqβgluc ranging between 8.43 and 11.43 mM and *V*_max_ values between 1,445.09 and 1685.00 U/mg were determined through Lineweaver-Burk and Michaelis–Menten plots, respectively. The *k*_cat_ value was found to be 454.05 min^−1^ for pPIC-Bteqβgluc and 448.72 min^−1^ for pET-Bteqβgluc, and the efficiency constant (*k*_cat_/ *K*_m_) was estimated to be 61.12 for pPIC-Bteqβgluc and 53.24 for pET-Bteqβgluc, indicating high catalytic power (conversion of bound substrate to product) of the enzyme.

**Figure 8:**
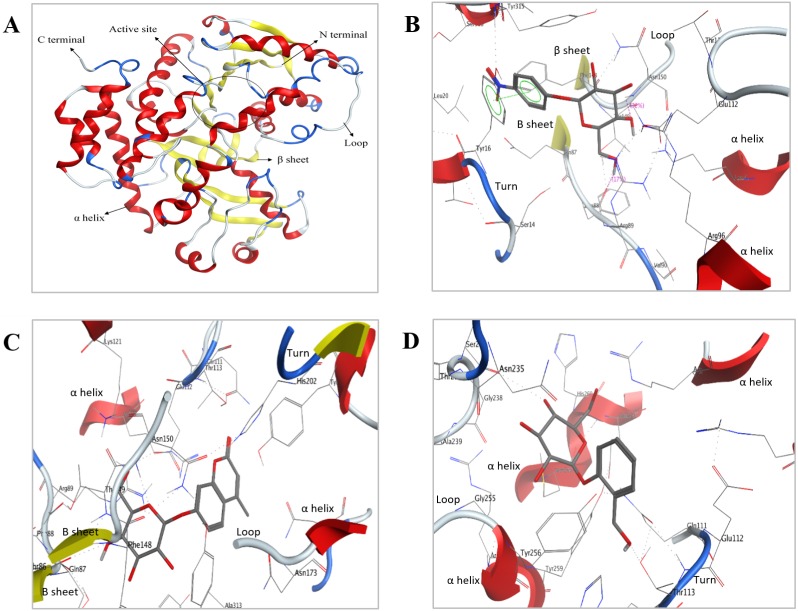
Predicted 3D ribbon structures of Bteqβgluc and substrates. Predicted 3D ribbon structure of Bteqβgluc (A). Representation of protein ligand interaction of docking complexes for (B) Bteqβgluc-pNPG, (C) Bteqβgluc-MUG, and (D) Bteqβgluc-salicin.

### Molecular docking

The QMEAN score and Ramachandran’s plot predicted a good quality model with >90% of residues in the most favored regions (Fig. S3), indicating an appropriately modeled structure. All other validation results of the SAVES Package (PROCHECK, and VERIFY3D) indicated that the predicted model was of good quality and could be further used for docking studies.

The predicted structure with minimized energy (Fig. 8A) was used for docking studies with *ρ*-nitrophenyl-linked substrates using MOE software. The best docking complex was used to analyze the enzyme-substrate interactions and to determine the conserved amino acid residues in the predicted catalytic cleft/active site. Figure 8B shows hydrogen-bonding interactions of Bteqβgluc with pNPG involving Glu112, Asn150, Phe88 and Tyr16 residues, expected to play an essential role in hydrolysis (2D presentation shown in Fig. S4). Surrounding amino acids Thr113, Phe148, Lys121, Arg89, Thr149, Leu20, Ser316, Tyr315 and Ala313 provide a hydrophobic environment enhancing enzyme activity and thermostability. MUG molecules showed hydrogen-bonding interactions with Bteq*β*gluc involving Asn150, Thr113 and Gln87 while, Ser14, Phe88, Thr149, Asn235, Arg89, Phe148, Lys121, His202, Tyr256, Gln111, Glu112, Tyr315 and Ala313 provide hydrophobic surrounding environment as showed in Fig. 8C (for 2D presentation). Hydrogen-bonding interactions of salicin involved Asn235 and Tyr16 residues of Bteqβgluc, with Met233, Tyr256, Glu112, Asn150, Thr113, His202, Tyr315, Arg89, Phe148, Ala313 and Val284 as surrounding residues providing stability to the complex as showed in Fig. 8D Fig. S4) for 2D presentation).

## Discussion

β-glucosidases are widely distributed and play pivotal roles in many biological processes, depending on the location of the enzyme and the biological system in which they occur (Ahmed et al., 2017; Singh, Verma & Kumar, 2015). The identification and characterization of *β*-glucosidases, their catalytic properties, and potential biological functions is of interest to discover enzymes with desirable properties for industrial application. Recombinant β-glucosidases have been previously produced with high yield in diverse heterologous systems.

In the current study, we cloned, expressed and characterized a β-glucosidase gene (Bteqβgluc) from *B. tequelensis* BD69 belonging to the GH4 family. Multiple sequence alignment supported maximum homology to Bacillus sp. CMAA 1185 and amino acid sequence similarity to β-glucosidases from different *Bacillus* species. The molecular weight of Bteq βgluc was also close to previously reported *β*-glucosidases from *Bacillus* species (Choi et al., 2009; Paavilainen, Hellman & Korpela, 1993; Zahoor et al., 2011). Existence of a predicted signal peptide suggests that Bteq βgluc is a secreted enzyme, as most of *Bacillus* spp. have a natural signal peptide secretion machinery (Westers, Westers & Quax, 2004).

Comparison between the bacterial and yeast recombinant Bteq*β*gluc showed higher protein yields and specific enzyme activity in the yeast system, as previously reported for a fungal chitinase β-glucosidase by Fan et al. (2007). These higher yields in yeast are related to accumulation of Bteqβgluc in insoluble inclusion bodies in *E. coli* cultures, comparatively reducing the levels of purified soluble protein even after solubilization and refolding. Differences in specific activity (U/mg) may be related to the different purification processes used for recombinant Bteqβgluc of bacterial and yeast origin or may be due to partial refolding of Bteqβgluc. Based on our results, expression of Bteqβgluc in yeast with multi-copy inserts should be the method of choice for production of considerable amounts of highly active enzyme for potential industrial applications.

The physical and kinetic reaction parameters of Bteqβgluc suggest some unique features for this enzyme. The p*K*_a_ or ionization constant, which describes the dependence of enzymatic activity on the pH of a reaction, depends on the ionization of essential active-site amino acid residues involved in substrate binding and catalysis (Niaz et al., 2004). The majority of β-glucosidases have p*K*_a_1 values between 3.3 and 4.4, and p*K*_a_2 between 6.1 and 6.9 (Clarke, Bray & Strating, 1993; Zahoor et al., 2011). Our preliminary tests suggest that Bteqβgluc may have a higher p*K*_a_1 (4.8) and p*K*_a_2 (6.4), which could evidence an ability to function under a broad pH range that includes weak alkaline conditions. More exhaustive determination of activity at multiple pH environments would be needed to obtain a more accurate estimate of p*K*a values.

The Bteqβgluc enzyme was thermostable (>30% activity remaining) between 30 °C and 60 °C, with optimum temperature for pNPG hydrolysis around 50 °C. The effect of temperature on the rate of reaction is also expressed in terms of *Q*_10_, which is the factor by which reaction rate increases due to a rise in temperature by 10 °C (Reyes, Pendergast & Yamazaki, 2008). The *Q*_10_ value for Bteq*β*gluc was similar to a β-glucosidase from white rot fungi (Mfombep, Senwo & Isikhuemhen, 2013) and within the thermostability range reported for most β-glucosidases, but lower than the maximum activity at 65 °C described for enzymes from *Fusarium oxysporum* (Christakopoulos et al., 1995).

Affinity for the substrate by Bteqβgluc, estimated with the Michaelis constant (K_m_) as an inverse measurement of affinity, was considerably higher (signifying lower affinity) compared to β-glucosidases from *Aspergillus niger* (Javed et al., 2018), *Methylococcus capsulatus* (Sathe et al., 2017), *Thermotoga naphthophila* (Akram, Iu & Mukhtar, 2018) or *Bacillus licheniformis* (Zahoor et al., 2011) or *Fusarium solani* (Bhatti, Batool & Afzal, 2013). However, the Bteqβgluc enzyme also displayed very high catalytic efficiency (*V*_max_) compared to similar enzymes (Table 2), suggesting higher conversion rate of substrate into product. This observation is also supported by lower energy of activation (*E*_a_), parameter describing the energy level that an enzyme must overcome before a reaction occurs, for Bteq*β*gluc when compared to β-glucosidase from *B. licheniformis* (Zahoor et al., 2011) or *Fusarium solani* (Bhatti, Batool & Afzal, 2013). In fact, the calculated values for the free energy of transition state binding (Δ*G*‡ (_E−T_) and free energy of substrate binding Δ*G*‡_E−S_) indicate high reactivity of the Bteqβgluc enzyme during pNPG hydrolysis. Other reaction parameters for pNPG hydrolysis by Bteqβgluc suggest a spontaneous (positive change in Gibbs free energy or Δ*G*‡) and endothermic reaction (positive enthalpy of activation or Δ*H*‡ and negative entropy of activation or Δ*S*‡). In comparison, Zahoor et al. (2011) reported higher values of Δ*H*‡ (64.04 kJ/mol) and Δ*S*‡ (48.28 J/mol/K) for *β*-glucosidase from *B. licheniformis*.

**Table 2:** Comparison of activity parameters among selected beta glucosidases. A comparison of physical and kinetic parameters determined for β-glucosidases of natural (N) and cloned (C) form from various microbial species. Units for *k*_cat_/*K*_m_ are as provided for *k*_cat_ and *K*_m_.

**Organism**	**Form**	**Optimum Temp & pH**	**Kinetic parameters**										**References**
			***V***_**max**_	***K***_**m**_(mM)	***k***_**cat**_(min^−1^)	***k***_**cat**_**/*K***_**m**_	**p*K***_**a**_**1**	**p*K***_**a**_**2**	***E***_**a**_**(kJ/mol)**	Δ***H***^‡^**(kJ/mol)**	Δ***G***^‡^**(kJ/mol)**	Δ***S***^‡^**(kJ/mol/K)**	
*Bacillus tequelensis* BD69- pPIC-Bteqβgluc	C	50 °C & 5.0	1,462.25 U/mg	7.43	454.05	61.12	4.8	6.4	44.18	41.69	57.88	−54.29	Present study
*Bacillus tequelensis* BD69- pET-Bteqβgluc	C	50 °C & 5.0	1,445.09 U/mg	8.43	448.72	53.24	4.8	6.4	45.29	42.82	57.85	−50.46	Present study
*Aspergillus niger*	N	–	–	0.24	43.15	59.71	–	–	50	–	–	–	Javed et al. (2018)
*Methylococcus capsulatus*	C	70 °C & 6.0	0.025 10^3^ U	0.12	3,330.5	27,597.0	–	–	–	–	–	–	Sathe et al. (2017)
*Thermotoga naphthophila*	C	85 °C & 5.0	153 × 10^3^U/mg	0.45	20,238.08	2,698,413	–	–	27.07	24.09	46.55	−62.74	Akram, Iu & Mukhtar (2018)
*Thermotoga naphthophila* RKU-10	C	95 °C & 7.0	297 × 10 ^3^U/mg	1.5	25,462.97	–	–	–	28.74	25.7	47.24	−58.6	Akram et al. (2016)
*Aspergillus niger*	N	50 °C & 5.0	166 U/mg	8	–	–	–	–	–	–	–	–	Narasimha et al. (2016)
*Bacillus sp.* SJ-10	C	40 °C & 6.0	1.526 U/mg	4.6	13.6 × 10^5^	3.0 × 10^5^	–	–	–	–	–	–	Lee et al. (2015)
*Thermotoga maritima*	C	80–100 °C & 5.0–7.0	238 ± 2.4 U/mg	0.56	3.12	–	–	–	36.92	33.73	127.96	−246.46	Mehmood et al. (2014)
*Bacillus subtilis* strain PS	C	60 °C & 5.0	0.94 U	–	6.92 ×10 ^−6^	–	–	–	–	–	–	–	Keerti et al. (2014)
white rot fungi	N	60 °C–70 °C & 3.5–5.0	0.21 μ g/min (*T. versicolor*) 9.70 μ g/min (*G. frondosa-28*)	0.47 × 10^−3^(*A. auricular*-1120)719 × 10^−3^(*L. edodes*-7)	–	–	–	–	–	–	–	–	Mfombep, Senwo & Isikhuemhen (2013)
*Fusarium solani*	N	65 °C & 4.5	55.6 U	1	–	–	–	–	53.31	–	–	–	Bhatti, Batool & Afzal (2013)
*Bacillus licheniformis*	C	50 °C & 6.0	1.26 U/mg	0.206	–	–	5.5	7	66.31	64.04	–	48.28 ×10^−3^	Zahoor et al. (2011)
*Bacillus subtilis* Natto	C	45 °C & 6.0	1.52 U/mg	–	0.118	3.01	–	–	–	–	–	–	Kuo & Lee (2008)
*Melanocarpus sp. MTCC 3922*	N	60 °C & 6.0	43.68 U/mg	3.3	4 × 10^3^	–	–	–	–	–	–	–	Kaur et al. (2007)
*Cellulomonas biazotea*	N	52 °C & 7.0	–	–	–	–	–	–	88	108	–	86 ×10^−3^	Rajoka et al. (2015)
*Cellulomonas biazotea* NIAB 442	N	38 °C & 6.6	0.22 U/mg	4.25	–	–	–	–	65	–	–	–	Siddiqui et al. (1997a)

Molecular docking was used as a tool to predict interactions between substrate and Bteqβgluc enzyme at the atomic level, helping to determine amino acid residues potentially involved in catalysis. This information can guide the design of site mutants to increase catalytic and functional characteristics. Our docking observations suggest involvement of Glu112, Asn150, Phe88 and Tyr16 residues in formation of the Bteq*β*gluc-pNPG complex, with Glu112 and Phe88 acting as backbone and side chain acceptors, respectively, while Asn150 acts as side chain donor and Tyr16 shows non-covalent interactions with the aromatic ring. These findings are in-line with previous studies (Haq et al., 2012; Rajoka et al., 2015). For instance, Tiwari et al. (2012) reported Glu as essential residue in the substrate binding pocket of β-glucosidase from *Cellulomonas biazotea*. In MUG-Bteqβgluc interactions, polar residues Asn150 and Thr113 form hydrogen bonds and act as backbone and side chain donors, respectively, while, Gln87 acts as backbone acceptor. In Bteqβgluc-salicin interactions, Asn235 and Tyr16 act as side chain donors. PLIF analysis identified Asn as a common active residue involved in catalysis among the above mentioned substrate-enzyme complexes.

## Conclusions

Data from the present study support higher production yields and specific activity for Bteqβgluc from *P. pastoris* compared to a bacterial expression system. The Bteqβgluc enzyme represents the first described β-glucosidase from *Bacillus tequelensis* and presents higher reactivity and processing rate, while maintaining thermostability and displaying activity under a wider range of pH conditions, when compared to alternative microbial β-glucosidases. Based on these observations, Bteqβgluc may have potential for industrial applications. Molecular docking analysis identified important residues for enzyme-ligand interactions that may be targeted in the future for catalytic or stability improvement.

##  Supplemental Information

10.7717/peerj.8792/supp-1 Supplemental Information 1 Supplementaryt figures Figures S1 to S6

10.7717/peerj.8792/supp-2 Supplemental Information 2 Raw data Raw data used for the preparation of the Dixon, Arrhenius and Lineweaver-Burk plots.

10.7717/peerj.8792/supp-3 Supplemental Information 3 Gene sequence for beta-glucosidase from *Bacillus tequelensis* Full length gene sequence of the beta-glucosidase characterized in the manuscript. GenBank accession (available after publication) MK774666.

## Additional Information and Declarations

### DNA Deposition

The following information was supplied regarding the deposition of DNA sequences: The gene reported here is GenBank accession number MK774666. The full length gene sequence is also provided as a Supplementary File. The protein sequences is shown in Fig. 1.

### Data Availability

The following information was supplied regarding data availability: The raw measurements of specific activity used for the Lineweaver-Burk, Arrhenius, and Dixon plots, mean and SD measurements for relative enzymatic activity and pH and thermal stability are all available in the Supplementary File.
