# Supplementary material for: Identification and functional characterization of a β-glucosidase from Bacillus tequelensis BD69 expressed in bacterial and yeast heterologous systems
Source: PeerJ. 2020 Mar 30;8:e8792. doi: 10.7717/peerj.8792 (PMC7115751; doi:10.7717/peerj.8792)
Supplement: Supplemental Information 1 — Figures S1 to S6 [file peerj-08-8792-s001.pdf]

PEERJ

Molecular docking and functional characterization of β-glucosidase from *Bacillus tequelensis* BD69 expressed in bacterial and yeast heterologous systems

Ahmad Raza[1,2,3], Ratnasri Pothula[3], Heba Abdelgaffar[3], Saira Bashir[1,2] and Juan Luis Jurat-Fuentes[3]

[1]National Institute for Biotechnology and Genetic Engineering (NIBGE), P.O. Box 577, Jhang Road, Faisalabad, Pakistan

[2]Pakistan Institute of Engineering and Applied Sciences (PIEAS), Islamabad, Pakistan

[3]Department of Entomology and Plant Pathology, University of Tennessee, Knoxville, TN 37996, USA.

Corresponding authors:

Saira Bashir,

sairabashir@gmail.com

Juan Luis Jurat-Fuentes,

jurat@utk.edu

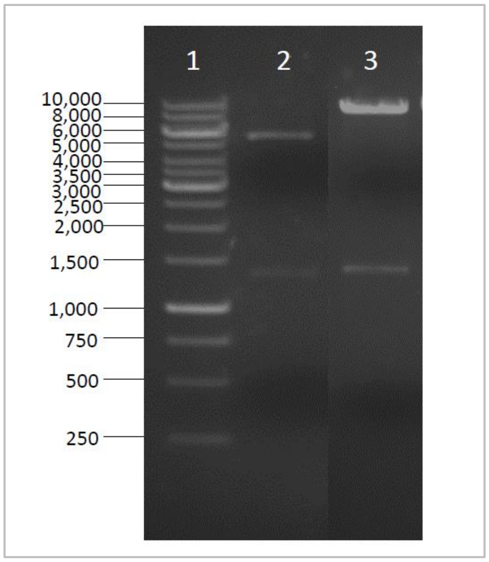

Fig S1. Double digestion of positive clones with EcoRI and NotI; Lane 1: GeneRuler 1-kb DNA ladder (Thermo Scientific); Lane 2: double digested pET-Bteqβgluc; Lane 3: double digested pPIC-Bteqβgluc.

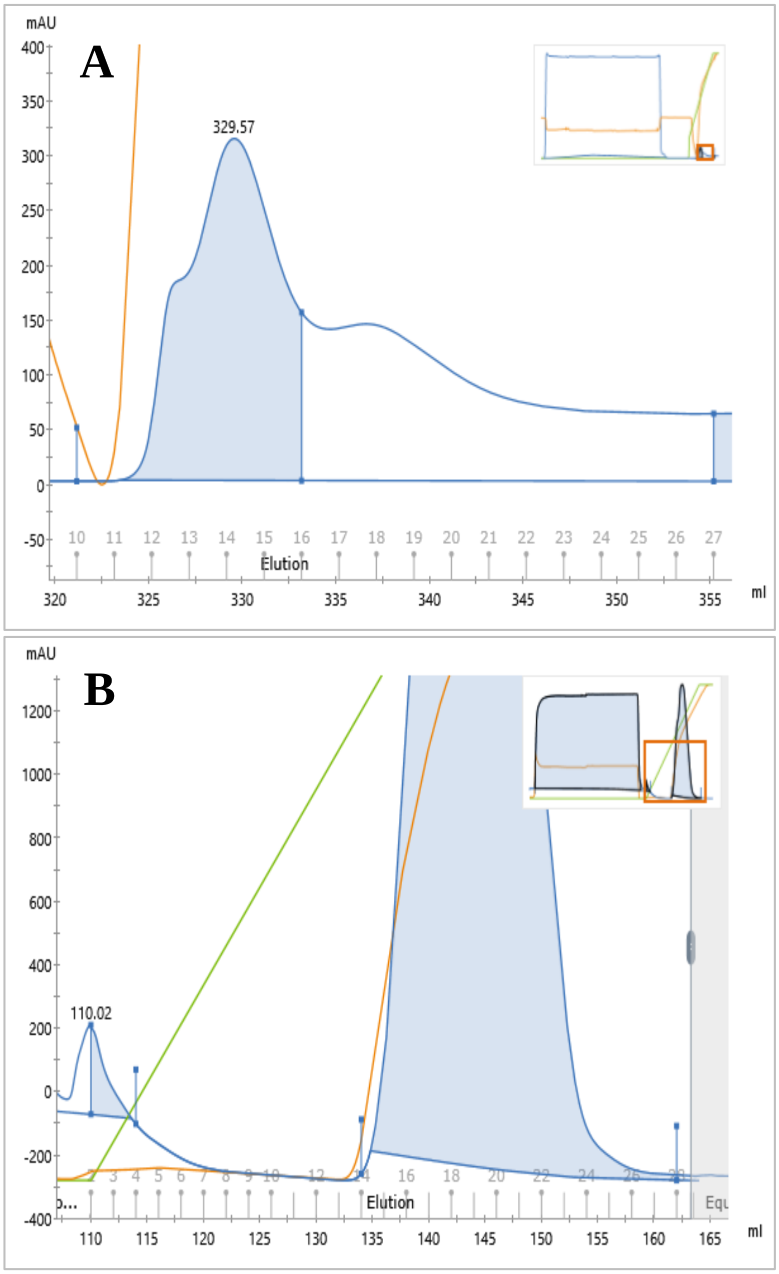

Fig S2. Chromatogram of β-glucosidase purification from AKTA Pure FPLC system (A) pET-Bteqβgluc (B) pPIC-Bteqβgluc.

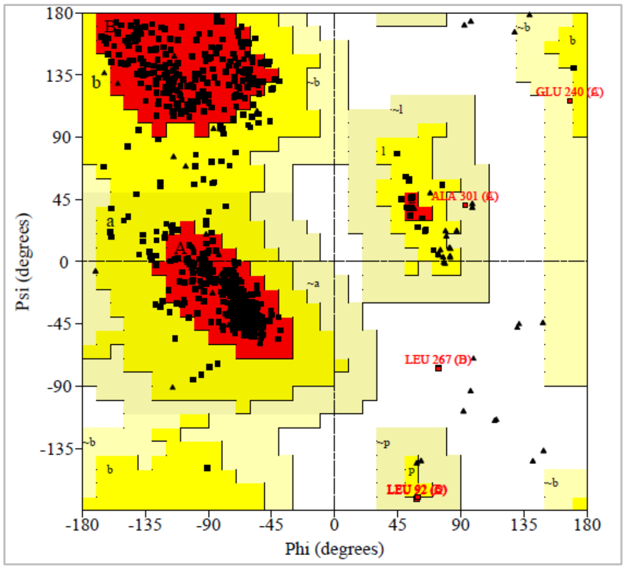

Fig S3. Ramachandran’s plot with >90% residues in the most favored regions for structural quality assessment of predicted Bteqβgluc structure.

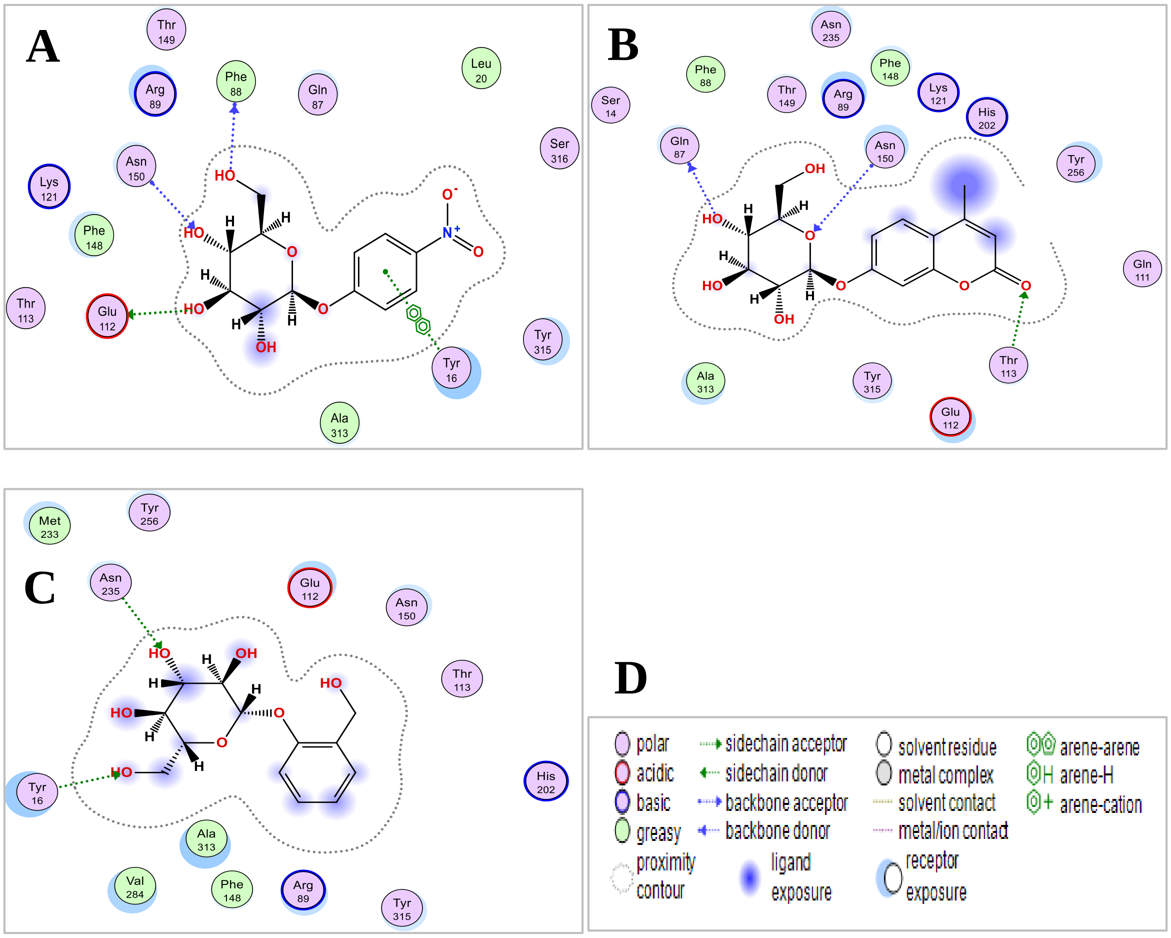

Fig. S4. Two dimensional representation of protein ligand interaction of docking complexes for (A) Bteqβgluc-pNPG, (B) Bteqβgluc-MUG, (C) Bteqβgluc-salicin and (D) legends of interaction diagram.

A

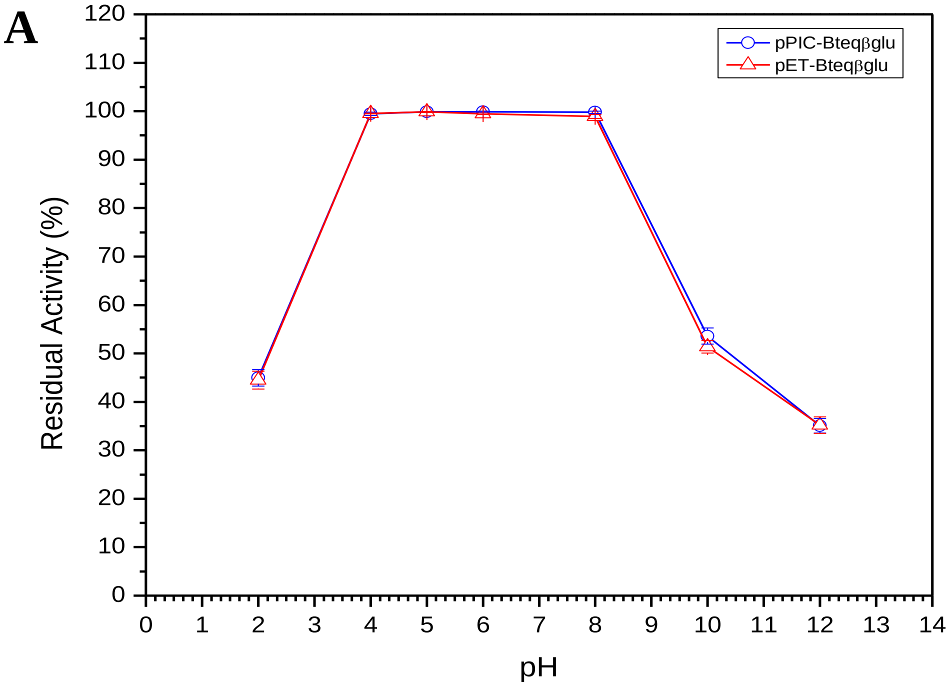
pPIC-Bteqβglu
pET-Bteqβglu
Residual Activity (%)
pH
0
10
20
30
40
50
60
70
80
90
100
110
120
0
1
2
3
4
5
6
7
8
9
10
11
12
13
14

B

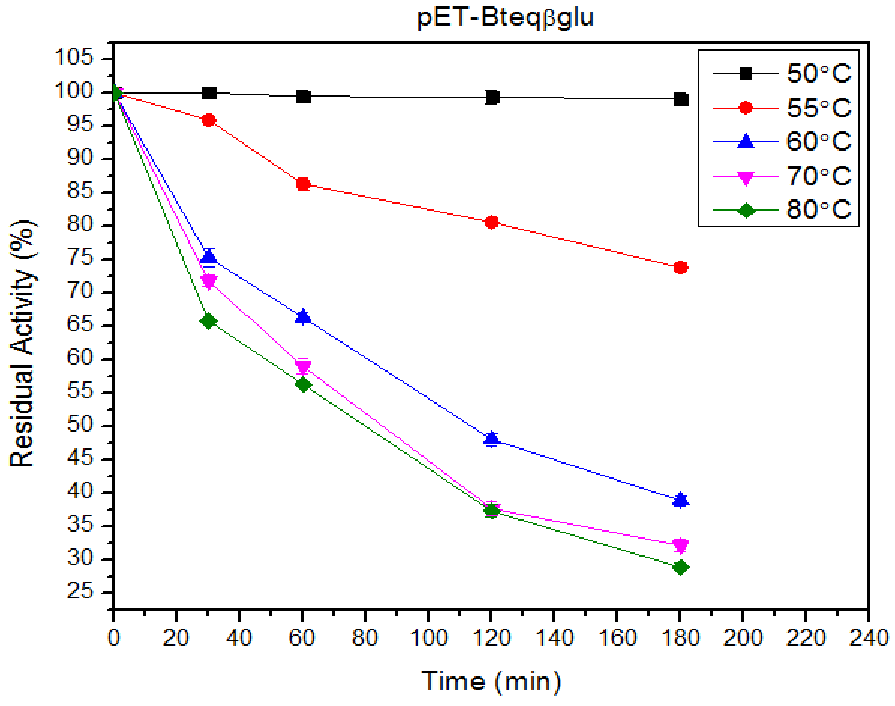
pET-Bteqβglu
50°C
55°C
60°C
70°C
80°C
Residual Activity (%)
Time (min)
25
30
35
40
45
50
55
60
65
70
75
80
85
90
95
100
105
0
20
40
60
80
100
120
140
160
180
200
220
240

C

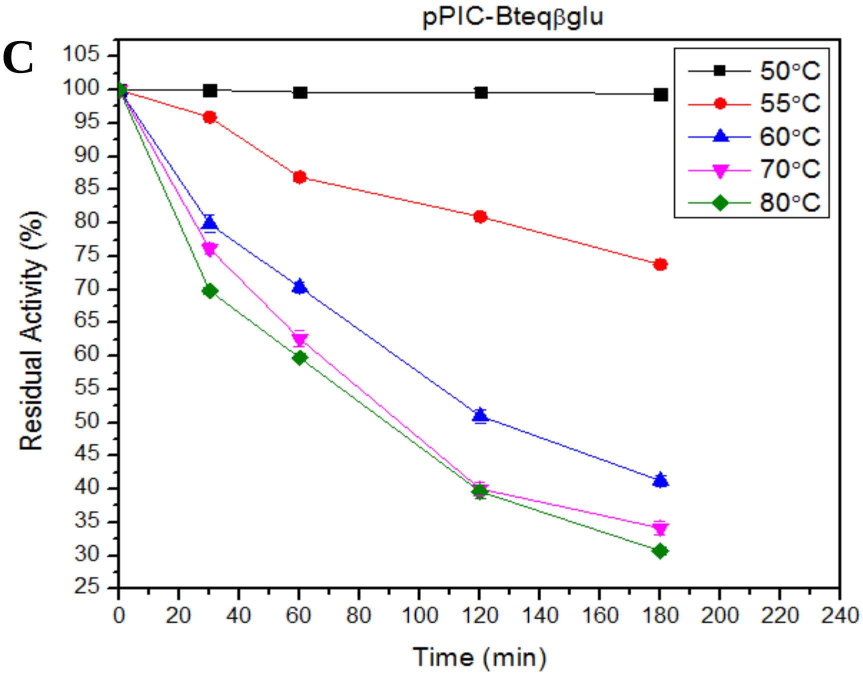

Fig. S5. pH stability (A) of pET-Bteqβgluc and pPIC-Bteqβgluc enzyme determined by pre-incubating at different pH values (2-12) at 50°C for 1 h and thermal-stability of pET-Bteqβgluc (B) pPIC-Bteqβgluc (C) was determined by pre-incubating enzyme at different temperature ranging from 50-80°C.

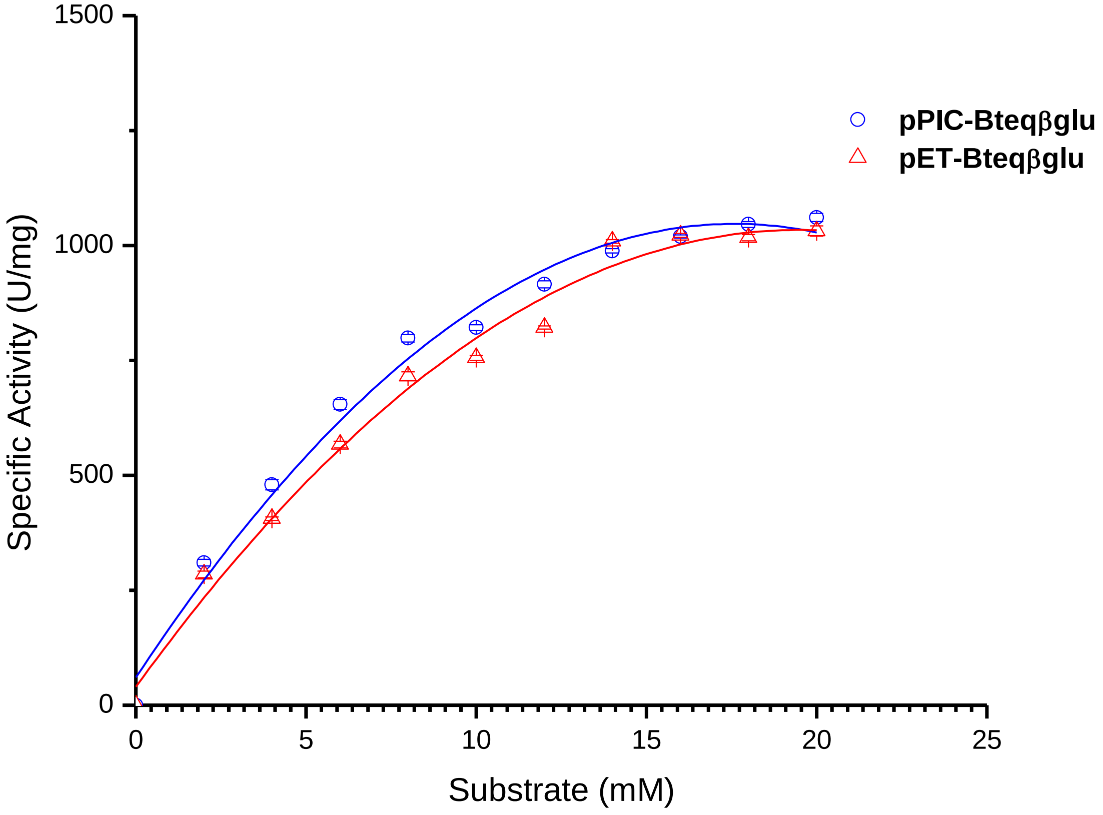

Fig. S6. Non-linear Michaelis-Menten plot of initial reaction velocity (U/mg) versus substrate concentration (mM) at a constant enzyme concentration for pPIC-Bteqβgluc and pET-Bteqβgluc.

Table S1. Summary of Two-way ANOVA test showing interaction effects within and between different substrate concentrations (2-20 mM) and enzyme culture groups (pET-Bteqβgluc and pPIC-Bteqβgluc) for specific activities.

Dependent Variable: Specific activities

Source	Type III Sum of Squares	df	Mean Square	F	Sig.
Corrected Model	3792862.188[a]	19	199624.326	3719.180	.000
Intercept	37088675.270	1	37088675.270	690995.264	.000
Groups (pET & pPIC)	32073.775	1	32073.775	597.563	.000
Substrate concentrations	3739327.880	9	415480.876	7740.781	.000
Groups * Sub. con.	21460.534	9	2384.504	44.425	.000
Error	2146.971	40	53.674		
Total	40883684.430	60			
Corrected Total	3795009.160	59			

a. R Squared = .999 (Adjusted R Squared = .999)

Table S2. Summary of Two-way ANOVA test showing interaction effects within and between different temperatures (30-80°C) and enzyme culture groups (pET-Bteqβgluc and pPIC-Bteqβgluc) for specific activities.

Dependent Variable: Specific activities

Source	Type III Sum of Squares	df	Mean Square	F	Sig.
Corrected Model	6102759.597[a]	17	358985.859	4425.912	.000
Intercept	22733965.084	1	22733965.084	280285.524	.000
Groups (pET & pPIC)	37890.236	1	37890.236	467.146	.000
Temperature	6043385.327	8	755423.166	9313.561	.000
Groups * Temp.	21484.034	8	2685.504	33.109	.000
Error	2919.961	36	81.110		
Total	28839644.641	54			
Corrected Total	6105679.558	53			

a. R Squared = 1.000 (Adjusted R Squared = .999)

Table S3. Summary of Two-way ANOVA test showing interaction effects within and between different pH (4-10) and enzyme culture groups (pET-Bteqβgluc and pPIC-Bteqβgluc) for Log10 V_{max}.

Dependent Variable: Log10 V_{max}

Source	Type III Sum of Squares	df	Mean Square	F	Sig.
Corrected Model	.795[a]	7	.114	1051.464	.000
Intercept	192.927	1	192.927	1786144.502	.000
Groups (pET & pPIC)	.004	1	.004	37.215	.000
pH	.777	3	.259	2396.707	.000
Groups * pH	.014	3	.005	44.303	.000
Error	.002	16	.000		
Total	193.724	24			
Corrected Total	.797	23			

a. R Squared = .998 (Adjusted R Squared = .997)
