# Supplementary material for: Identification and functional characterization of a β-glucosidase from Bacillus tequelensis BD69 expressed in bacterial and yeast heterologous systems
Source: PeerJ. 2020 Mar 30;8:e8792. doi: 10.7717/peerj.8792 (PMC7115751; doi:10.7717/peerj.8792)
Supplement: Supplemental Information 3 — Full length gene sequence of the beta-glucosidase characterized in the manuscript. GenBank accession (available after publication) MK774666. [file peerj-08-8792-s003.pdf]

> Beta-glucosidase from *Bacillus tequilensis* (Bteqβgluc; MK774666)

ATGACAAAAGGATTGAAGATTGTAACGATTGGCGGAGGTTCAAGCTATACGCCCGTGCTAGTGGAAGGGTTCATT
AAACGGTATGATGAGCTGCCTGTGCGTGAATTATGGCTCGTCGATATCCCTGAAGGCGAAGAAAAGCTGAATATT
GTCGGCACACTCGCCAAACGGATGGTTGAAAAAGCAGGCGTCCCGATCGACATTCATCTTACGCCCGATCGGAGA
AAAGCGCTGAAAGACGCAGACTTTGTTACGACTCAATTCCGTGTCGGACTTTTGCAGGCAAGAGCAAAGGATGAG
CGCATTCCACTAAAATACGGAGTAATTGGCCAGGAAACAAACGGTCCGGGAGGCCTGTTTAAAGGATTACGCACG
ATTCCGGTCATCCTTGAGATCGCAAAGGATATAGAAGAGCTCTGCCCGAACGCTTGGCTTGTTAACTTCACAAACC
CTGCCGGCATGGTAACAGAAGCCTTGCTTCGCTACTCCAACCTGAAGAAAGTCGTCGGTCTTTGTAATGTTCCAAT
CGGCATTAAAATGGGCGTAGCCAAAGCGCTTGATGTAGATGTGGACCGCGTAGAAGTCCAATTCGCCGGACTGAA
TCATATGGTGTTCGGGCTGGATGTTTTCTTGGACGGCGTAAGTGTGAAAGAGCAAGTCATCGAGGCGATGGGTGA
CCCGAAAAACGCGATGACGATGAAAAATACCTCAGGCGCCGAATGGGAACCGGATTTCTTAAAAGCGCTCAATGT
GATCCCTTGCGGCTATCACCGTTACTATTTCAAAACAAAAGAAATGCTGGAGCACGAACTGGAAGCATCACAAACG
GAGGGCACCCGCGCTGAAGTCGTGCAAAAGGTCGAAAAAGAGCTGTTTGAGCTCTATAAAGATCCGAACCTTGCG
ATCAAACCGCCGCAGCTGGAAAAACGGGGCGGAGCTTACTACAGTGATGCAGCATGTAACCTAATCAGCTCCATT
TACAATGATAAACATGACATTCAGCCGGTGAACACGATCAATAATGGTGCAATAGCGAGCATTCCGGACGATTCC
GCAGTTGAAGTGAACTGTGTGATGACGAAAACAGGCCCTAAGCCAATTGCTGTCGGCGACTTGCCGGTGTCTGTC
CGAGGCCTCGTCCAGCAAATCAAATCCTTTGAGCGTGTCGCAGCAGAAGCGGCAGTTACAGGTGACTATCAAACC
GCCCTCCTTGCCATGACGACTAACCCGCTTGTCCCGTCCGATGCTGTGGCCAAACAGATTTTAGACGACATGCTGG
AGGCGCACAAAGCGTATCTGCCGCAGTTTTTCAACAAAATTGAAGCGTAA
